# Improving rural health services: The case of a family physician’s contribution at Aber Hospital, Northern Uganda

**DOI:** 10.4102/phcfm.v13i1.3229

**Published:** 2021-12-13

**Authors:** Samuel Okori, Innocent K. Besigye

**Affiliations:** 1 St. John XXIII Hospital-Aber, Lira, Oyam District, Uganda; 2 Department of Family Medicine, College of Health Sciences, Makerere University, Kampala, Uganda

**Keywords:** family medicine, rural health, primary health care, primary care, health systems

## Abstract

Family physicians (FPs) provide quality comprehensive primary care services responsive to the needs of the people they are serving. In Uganda, FPs are still few with poor visibility hence difficult to demonstrate their impact. This short report describes the contribution of a FP guided by the principles of family medicine to improving health care services to meet the needs of a rural population in Northern Uganda. This was carried out through targeted capacity building for teams within various hospital departments and the provision of transformative leadership and management. Hospital laboratory and radiology departments were strengthened to provide the needed diagnostic services to the population and human immunodeficiency virus (HIV) care and tuberculosis screening were improved through the establishment of community service centres together with strengthening community outreaches. The transformative leadership of the multidisciplinary team provided by the FP significantly improved the quantity and quality of health care services.

## Background

Family physicians (FPs) are trained to provide quality primary care services that are responsive to the health needs of individuals, families and communities. They perform this function according to the principles of family medicine (FM).^1,2,3^

Family physicians contribute to improving the performance of health systems.^4,5^ In Uganda, FPs perform several roles including clinical care, leadership of health care teams, manager of human and capital resources as well as teaching health professions students.^5^ They are able to perform these roles because of their training as stewards of the health care system that aims to improve the quantity and quality of the services.

As in most other African countries, FM is a relatively new discipline in Uganda with small numbers of FPs in practice.^6^ Therefore, the visibility of the speciality is poor with limited opportunities to demonstrate its impact. The speciality is not well known with few doctors choosing it as their career choice. This makes it difficult for policymakers and decision makers to see the benefits of well-trained generalist FPs. There is a need to document and showcase the contributions of the few FPs practising in Uganda. This short report describes the contribution of an FP based at Aber Hospital to improving rural health care in Northern Uganda.

## Context

Aber Hospital, a 220-bed faith-based private not-for-profit hospital, with a catchment population of about 51 000 people, is located in rural Northern Uganda. It is a general hospital that also offers comprehensive primary care. The hospital receives government support and subsidies, including primary health care (PHC) grants, to enable the provision of services at low cost. Northern Uganda is a post-conflict area, which suffered civil war for more than 20 years. As a result, there is a severe shortage of medical personnel of all cadres compared to other regions in Uganda. For example, most hospitals in the region have less than five medical officers serving large patient numbers and experience high attrition rates because of poor socio-economic conditions and infrastructure. Previously to specialist care was limited with many patients travelling to Kampala, the capital city, in search of health care services because of the lack of skilled medical personnel.

The hospital serves as a referral facility for lower level PHC facilities as it forms its apex. It provides curative general medical and surgical services including diagnostic services, health promotion and preventive services. The community served is mainly rural subsistence peasant farmers. The FP re-oriented the emphasis to PHC focusing on health promotion, disease prevention and aligning the services provided to the needs of the people. Outside the hospital are lower level PHC services from Health centre I also called Village Health Team, then Health Centre II, Health Centre III, Health Centre IV and then referral is made to the general hospital that offers PHC but also serves as a referral PHC facility.

## Contribution

The first author started working at Aber Hospital in 2016 after completion of his postgraduate family medicine training in 2013. He embarked on improving the services offered by the hospital within and outside the hospital. Priority areas were identified and special efforts instituted to initiate new services and improve the performance of other areas. This was done with the involvement of both internal and external stakeholders. Capacity development of the hospital teams in the various departments was key, with the belief that a motivated, skilled and competent workforce would be the main driver for improved performance. Relevant short courses were identified and training was targeted towards clinical officers, nurses, midwives and laboratory personnel.

The FP engaged with the following activities to improve the services:

- Organisation of regular community outreach visits by the hospital’s clinical staff to improve immunisation coverage, access to human immunodeficiency virus (HIV) testing and tuberculosis screening, maternal and child health care and referrals from the community. Community outreach visits involved nurses, doctors and midwives going out to communities with essential drugs, vaccines and other supplies to provide health education, immunisations and medical services. The community was mobilised with the help of community health workers (CHWs) who were linked to the hospital. The community members in need of these services gathered at a designated place in the community where the outreach team could meet them.
- Equipping and expanding the laboratory to improve the services. An application for accreditation was then made and it is now the only internationally accredited laboratory in the region.
- Improvement of the radiology department by procurement of a computer tomography (CT) scanner, the first and only CT scan machine in this region.
- Capacity building of the hospital staff and outreach teams through regular continuous professional development sessions and targeted short course trainings.
- Innovative HIV care and treatment by setting up 33 community distribution points for anti-retroviral (ARV) drugs. Each point serves an average of 70 patients monthly, which greatly improved the access to ARVs for people living with HIV from 2123 to 5200 over a 5-year period.
- Strengthening of linkage to care with emphasis on ambulance services. Regular engagement and communication with CHWs, encouraging them to identify and refer patients to the hospital. A hospital telephone line was given to the CHWs for emergency calls in case a patient needed to be transported by ambulance. A modest monetary contribution was made towards this service.

Table 1 shows the improvements in health care services as a result of the above actions.

**TABLE 1:** Improvement in health care services over a 5-year period (2015–2020).

Activity/number	Before family physician	After family physician
Community outreaches conducted per month	2	16
Patients visiting outpatient department	36 000	41 900
Patients attended in the laboratory	34 500	90 200
Patients attended the radiology department	2500	5200
Tests carried out the laboratory	25 308	54 796
Continuous professional development (CPD) sessions per year	48	144
Referrals into the hospital	250	1200
Referrals out of the hospital for specialised care	450	120
Average number of patients seen per community out-reach	8	70

As a result of these improvements, in-hospital neonatal mortality decreased from 53% to 10%, major surgeries performed increased from 530 to 1620, maternity admissions increased from 2700 to 3313 and babies delivered increased from 1872 to 2967.

## Reflection on the contribution of the family physician

The extensive knowledge and skills acquired during FM training helped the FP to improve care at the hospital and provide more comprehensive PHC in the community. The Improvement was linked to the direct clinical care from the FP as well as the capacity building of the whole health care team. Capacity building not only involved teaching and training to create a more competent and motivated workforce, but also included changes to the organisation of care and service delivery. Changes to the organisation of care required leadership and managerial skills. These changes required the collaboration and engagement of all clinical, support and managerial staff, but the FP was a critical catalyst in making these changes.

This short report illustrates the impact that a specialist in family medicine can make in under-resourced rural and remote areas. In Uganda, there is a need for more deliberate efforts to deploy at least two FPs in each general hospital in order to improve health care services.

## Conclusion

Improving rural health care requires a complex mixture of skills within a multidisciplinary team. Such a team will need the leadership of a well-trained generalist with clinical, educational and organisational knowledge and skills to improve the quality of service delivery and make the services more responsive to the needs of the people. Highly trained FPs strategically deployed within the health care delivery system can greatly improve the quantity and quality of health care services particularly in regions with limited access to specialist care. Uganda should scale up its deployment of FPs in PHC and general hospitals.
